# Exogenous Bimodal Cues Attenuate Age-Related Audiovisual Integration

**DOI:** 10.1177/20416695211020768

**Published:** 2021-05-27

**Authors:** Yanna Ren, Ying Zhang, Yawei Hou, Junyuan Li, Junhao Bi, Weiping Yang

**Affiliations:** Department of Psychology, College of Humanities and Management, Guizhou University of Traditional Chinese Medicine, Guiyang, China; Department of Psychology, Faculty of Education, 12563 Hubei University, Wuhan, China

**Keywords:** audiovisual integration, exogenous attention, bimodal cue, unimodal cue, older adults

## Abstract

Previous studies have demonstrated that exogenous attention decreases audiovisual integration (AVI); however, whether the AVI is different when exogenous attention is elicited by bimodal and unimodal cues and its aging effect remain unclear. To clarify this matter, 20 older adults and 20 younger adults were recruited to conduct an auditory/visual discrimination task following bimodal audiovisual cues or unimodal auditory/visual cues. The results showed that the response to all stimulus types was faster in younger adults compared with older adults, and the response was faster when responding to audiovisual stimuli compared with auditory or visual stimuli. Analysis using the race model revealed that the AVI was lower in the exogenous-cue conditions compared with the no-cue condition for both older and younger adults. The AVI was observed in all exogenous-cue conditions for the younger adults (visual cue > auditory cue > audiovisual cue); however, for older adults, the AVI was only found in the visual-cue condition. In addition, the AVI was lower in older adults compared to younger adults under no- and visual-cue conditions. These results suggested that exogenous attention decreased the AVI, and the AVI was lower in exogenous attention elicited by bimodal-cue than by unimodal-cue conditions. In addition, the AVI was reduced for older adults compared with younger adults under exogenous attention.

In daily life, individuals are often inundated with stimuli from multiple sensory modalities, including auditory, visual, somatosensory, olfactory, and gustatory stimuli. To acquire an appropriate perception of the outside world, our brain can effectively screen and integrate effective information out of the dynamic complex information coming from the environment. The process that merges information from auditory and visual modalities is called audiovisual integration (AVI) (Meredith et al., 1987; Spence, 2011; Stein, 2012; Stein & Meredith, 1993). Studies have revealed that responses to audiovisual stimuli are faster and more accurate than responses to unimodal auditory or visual stimuli (Miller, 1982; Raab, 1962). Attention is a key factor that alters the processing of sensory stimuli by enhancing the perception of the attended location (Ho et al., 2009; McDonald et al., 2000), and an effective cue is able to shift the attentional resource to a cued location and then facilitate the response to the subsequently presented stimuli at the cued location (McDonald et al., 2005; Mozolic et al., 2008; Spence, 2010). Exogenous attention, which is also called stimulus-driven or involuntary attention, can be induced reflexively by a salient sensory event in the external environment (Hopfinger & West, 2006; Tang et al., 2016). Studies have shown that the exogenous attention elicited by exogenous cues effectively captures an individual’s attention and is unable to be ignored (Berger et al., 2005). Van der Stoep et al. (2015) first investigated the interaction of exogenous attention and the AVI using a simple light/sound detection task. In their experiment, sight and sound were presented randomly with or without exogenous auditory cues, and the participant was instructed to respond to the stimuli as quickly and accurately as possible. Their results showed that the AVI was weaker in the exogenous cue condition compared with the no-cue condition regardless of the location of the light/sound stimuli. In their following study, the similar program was employed, but the visual cue was applied instead of the auditory cue, and the consistent conclusion was obtained that the AVI was lower under exogenous-cue condition than under no cue condition (Van der Stoep et al., 2017). Recently, Xu et al. (2020) also described a decreased AVI under exogenous auditory cues and further reported that the effect of exogenous auditory cues on the AVI was greatest when the cue-target onset asynchrony was 200 ms (Xu et al., 2020).

Serial studies by Spence et al. have demonstrated that the exogenous cueing effect can be evoked by both unimodal cues and bimodal cues, and the exogenous attention elicited by bimodal cues is different from that elicited by unimodal spatial cues (Santangelo & Spence, 2007; Santangelo et al., 2008; Spence, 2010; Spence & Driver, 1999; Spence & Santangelo, 2009). Santangelo et al. (2008) designed a visual spatial discrimination task to specifically assess whether the visual event-related potential component was the same when exogenous attention was evoked by exogenous unimodal auditory/visual cues and bimodal audiovisual cue, and no significant difference was noted in spatial orienting evoked by unimodal and bimodal cues (Santangelo et al., 2008). To further assess attentional strength and stabilization when evoked by unimodal and bimodal cues, a visual detection task with or without perceptual load was conducted (Santangelo & Spence, 2007). In the condition without perceptual load, the participant was instructed only to conduct a visual spatial discrimination task, but the participant was instructed to monitor a rapidly presented central stream of visual letters for occasionally presented target digits while responding to the visual detection task in the perceptual load condition. The researchers compared the ability of capturing visuospatial attention between exogenous unimodal cues (visual and auditory cues) and bimodal cues (audiovisual cue) and found that bimodal audiovisual cues captured participants’ spatial attention more effectively and stably than unimodal auditory or visual cues. Consistent results were also obtained in the bimodal audio-tactile cue conditions, illustrating that the spatial cueing effect was stronger when it was elicited by bimodal audio-tactile cues compared with unimodal auditory or tactile cues (Spence & Santangelo, 2008). In studies by Van der Stoep et al. (2015) and Xu et al. (2020), auditory cues were applied, and visual exogenous cues were applied in Van der Stoep et al.’s second experiment (Van der Stoep et al., 2017). Because the exogenous attention elicited by auditory cues and visual cues is not stable (Santangelo & Spence, 2007; Santangelo et al., 2008; Spence, 2010; Spence & Driver, 1999; Spence & Santangelo, 2009), the interaction between exogenous attention and AVI might be unreliable in conditions in which exogenous attention is elicited by unimodal cues (auditory or visual). In addition, considering that the exogenous attention elicited by bimodal cues is more effective and stable, it is necessary to evaluate the AVI when exogenous attention is elicited by bimodal cues to completely confirm the interaction between exogenous attention and AVI.

Therefore, the first interest of the present study was to investigate the different effects of exogenous bimodal cue (audiovisual) and unimodal cues (visual or auditory) on the AVI. Considering that bimodal cues could evoke higher and more stable cueing effects (Santangelo & Spence, 2007; Spence & Santangelo, 2008) and exogenous attention decreases the AVI (Van der Stoep et al., 2015, 2017; Xu et al., 2020), we hypothesized a weaker AVI in bimodal audiovisual cue condition compared with unimodal auditory or visual cue conditions.

In addition, compared with younger adults, the visual acuity was lower and the auditory threshold was higher in older adults (Cliff et al., 2013; Grady, 2009; Liu & Yan, 2007; Spear, 1993), and this deterioration leads to poorer health status and cognitive functional decline in older adults (Freiherr et al., 2013). Although some studies reported a decreased AVI for older adults compared with younger adults (Mahoney et al., 2011; Ren, Li, et al., 2020; Ren et al., 2016, 2018; Wu et al., 2012) resulting from age-related general cognitive functional decline, numerous studies also found a higher AVI for older adults than for younger adults (Deloss et al., 2013; Laurienti et al., 2006; Peiffer et al., 2007; Sekiyama et al., 2014; Zou et al., 2017). Neuroimaging studies further proposed that older adults might recruit new brain networks (Diaconescu et al., 2013; Ren et al., 2018; Ren, Li, et al., 2020; Ren, Xu, et al., 2020) and strengthen global brain connectivity (Wang et al., 2018) during audiovisual information processing, indicating that it is an adaptive mechanism. However, with aging, excepting for vision and audition disorders, there are serious attentional deficits (Kok, 2000; Plude et al., 1994; Quigley et al., 2010; Williams et al., 2016), showing that there are distractor suppression deficits in older individuals (Kok, 2000; Plude et al., 1994; Quigley et al., 2010), and older adults are much more susceptible to irrelevant distractors (Williams et al., 2016). Therefore, another interest of the present study was to investigate whether older adults could integrate auditory and visual information effectively under different exogenous attentional conditions.

## Methods

### Subjects

Twenty healthy older adults (59–76 years, mean age ± standard deviation [*SD*], 63.9 ± 4.8) and 20 young adults (19–25 years, mean age ± *SD*, 21.7 ± 1.4) participated in this study. All of the younger adults were college students at Guizhou University of Traditional Medicine, and the older adults were citizens of Guiyang City. All participants were free of neurological diseases, had normal or corrected-to-normal vision and were naïve to the purpose of the experiment. Participants were excluded if their Mini-Mental State Examination (MMSE) scores were greater than 2.5 *SD*s from the mean for their age and education level (Bravo & Hébert, 1997). In addition, participants who reported a history of cognitive disorder were excluded from the experiment. All participants provided written informed consent for the procedure, which was previously approved by the Second Affiliated Hospital of Guizhou University of Traditional Chinese Medicine. All participants were paid 60 RMB per hour for their time and completed the experiment successfully.

### Stimuli

**Figure 1.**
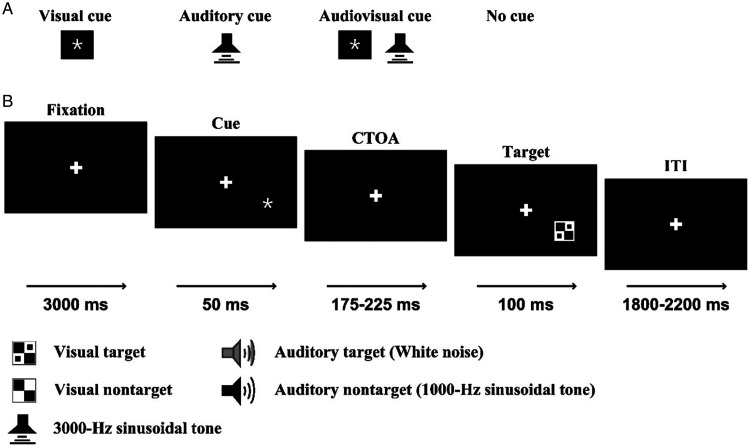
General layout of the experimental paradigm in this study. A: Cue type. B: A possible trial sequence for visual target under visual cue condition. CTOA = cue-target onset asynchrony; ITI = interstimulus interval.

The auditory nontarget stimulus was a 1000-Hz sinusoidal tone, and the auditory target stimulus was white noise (Ren et al., 2016, 2018; Yang et al., 2015). The visual nontarget stimulus was a black and white checkerboard image (B/W checkerboard, 52 × 52 mm, with a visual angle of 5°), and the visual target stimulus was a B/W checkerboard image with two black dots located within each white checkerboard (He et al., 1996; Laura et al., 2005; Ren et al., 2016). The audiovisual nontarget stimulus was the simultaneous presented visual nontarget stimulus and auditory nontarget stimulus, and the audiovisual target stimulus was the simultaneous presented auditory target stimulus and visual target stimulus. The following conditions were not included: a visual target stimulus accompanied by an auditory nontarget stimulus and a visual nontarget stimulus accompanied by an auditory target stimulus (Fournier & Eriksen, 1990). Besides, the visual stimulus and auditory stimulus always presented on the same hemifield, that is, the left visual stimulus was always companied with left auditory stimulus, and the right visual stimulus was always companied with right auditory stimulus. The visual stimuli (V) were presented on a computer monitor in front of participants’ eyes and on the lower left or right quadrant of the screen for 100 ms with a 12° visual angle (Figure 1B). The auditory stimuli (A) were presented through two speakers at approximately 60-dB SPL for a duration of 100 ms (Ho et al., 2009; Ren et al., 2016).

The unimodal auditory cue was 3000-Hz 60-dB sinusoidal tone for a duration of 50 ms and was presented on the speaker. The unimodal visual cue was a white asterisk and was presented at the same location as the visual stimuli. The bimodal audiovisual cue was the simultaneous presented auditory cue and visual cue on the same hemifield (Figure 1A). All of the auditory cue, visual cue, and audiovisual cue could be presented on the left hemifield or right hemifield randomly. If the cues and the following stimuli were presented on the same hemifield, it was defined as valid cue; otherwise, invalid cue.

### Procedure

The subjects were instructed to perform the experiment in a dimly lit, electrically shielded, and sound-attenuated room. Stimulus presentation and response collection were conducted using E-prime 2.0 software (Psychology Software Tolls, Inc., Pittsburgh, PA, USA).

The experiment contained the following three blocks: bimodal audiovisual cue block (AV_cue), unimodal visual cue block (V_cue), and unimodal auditory cue block (A_cue). At the beginning of each session, subjects were presented with a fixation cross for 3,000 ms. Following fixation, a cue (valid, invalid, or no cue) was selectively presented with equal probability (450 trials for each cue type) for 50 ms. Then, 40 trials for each target stimulus (A, V, AV) and 10 trials for each nontarget stimulus (A, V, AV) were presented randomly with a random time interval between cue and target (cue-target onset asynchrony, CTOA) from 175 ms to 225 ms. The interstimulus interval was randomly presented from 1,800 ms to 2,200 ms (Figure 1B). The participants were instructed to respond to target stimuli as accurately and quickly as possibly by pressing the button under the index finger of their right hand, but withhold their response for all nontarget stimuli. In total, three blocks were conducted with each task lasting approximately 15 minutes with appropriate rest with request of each subject. The order in which participants conducted the three blocks was randomized and counterbalanced across participants.

### Data Analysis

The accuracy, false alarm, and response time (RT) were computed separately for each subject under each condition, and then, the data were submitted to a group (Older, Younger) × Cue Type (A_cue, V_cue, AV_cue) × Cue Validity (Valid, Invalid, No cue) × Stimulus Type (A, V, AV) analysis of variance (ANOVA) separately (Greenhouse–Geisser corrections with corrected degrees of freedom).

The AVI was tested using individual cumulative distribution functions (CDFs) of each target type under each cue condition to compute race model as Formula 1 (Miller, 1982; Raab, 1962), basing on corrected response and omitted response (Miller, 2004, Appax A). P(RT_AV<_*_t_*), P(RT_A<_*_t_*), P(RT_V<_*_t_*) denotes the probability of responding within a given timeframe *t* in audiovisual, auditory, and visual stimulus condition, respectively. Besides, the *kill-the-twin*-correction was employed for wrong response of nontarget stimuli (Eriksen, 1988; Gondan & Minakata, 2015, Ineq 10).

(1) $$\text{P}\left({\text{RT}}_{\text{AV}<t}\right)\leq \text{P}\left({\text{RT}}_{\text{A}<t}\right)+\text{P}\left({\text{RT}}_{\text{V}<t}\right)$$

Miller (1982) proposed the race modal inequality that P_AV_ never exceeds (P_A_ + P_V_) for all *t*; therefore, the paired-permutation tests with 10,000 repeats was employed to examine whether the race model inequality was hold in each cue condition for each time point (*t*) (Gondan, 2010; Gondan & Minakata, 2015). If the response to AV (P_AV_) significantly violated race model inequality (P_A_ + P_V_), the AVI was assumed to have occurred (Gondan & Minakata, 2015; Miller, 1982). For each cue-type condition, the time interval of AVI was calculated separately, and then, the *t*_max_ statistic was employed across each time interval (Gondan, 2010; Gondan & Minakata, 2015).

To further assess the effect of cue-type on the amount of AVI, the positive area under the different inequality curve (pAUC) was also calculated for each participant (Colonius & Diederich, 2006; Van der Stoep et al., 2015). The negative area indicated obeyed the race model, and was set to zero, and only the positive area was calculated (Colonius & Diederich, 2006; Van der Stoep et al., 2015). And then, the individual pAUC was submitted to Group (Older, Younger) × Cue Type (A_cue, V_cue, AV_cue, No_cue) ANOVA (Greenhouse–Geisser corrections with corrected degrees of freedom) to evaluate the diversity of the AVI between older and younger adults and among different cue types.

## Results

### Accuracy and False Alarm

The accuracy was greater than 88% and the false alarm was lower than 15% for each stimulus under each condition for both older and younger adults (Table 1). The Group (Older, Younger) × Cue Type (A_cue, V_cue, AV_cue) × Cue Validity (Valid, Invalid, No cue) × Stimulus Type (A, V, AV) ANOVA for accuracy exhibit a significant main effects of group—*F*(1, 38) = 23.44, *p* < .001, indicating a higher accuracy for younger adults compared with older adults, and stimulus type—*F*(2, 76) = 14.16, *p* < .001, demonstrating a higher accuracy to AV stimuli compared with A or V stimuli (AV > A > V). The analysis for false alarm revealed a main effects of group—*F*(1, 38) = 9.10, *p* = .005, indicating higher false alarm for older adults than for younger adult, and stimulus—*F*(2, 76) =8.27, *p* = .001, demonstrating higher false alarm for V stimuli compared with A or AV stimuli (V > AV > A). No other significant main effect and interaction were found.

**Table 1.** Response Time, Accuracy, and False Alarm for Older and Younger Adults With the Standard Deviation in Each Condition.

		V_cue								A_cue									AV_cue								
	Valid			Invalid			No cue			Valid			Invalid			No cue			Valid			Invalid			No cue		
	A	V	AV	A	V	AV	A	V	AV	A	V	AV	A	V	AV	A	V	AV	A	V	AV	A	V	AV	A	V	AV
Response time (ms)																											
Older	558	518	466	577	552	503	646	569	517	566	480	456	570	507	469	651	546	512	541	486	459	548	520	476	681	575	532
SD	94	65	64	101	57	89	94	73	78	98	52	76	95	63	71	108	66	78	80	59	54	90	61	55	98	66	61
Younger	493	437	394	486	463	407	565	511	459	498	443	411	501	454	427	590	498	468	466	420	383	468	437	388	559	494	460
SD	92	87	74	94	92	85	100	79	75	67	64	79	63	66	89	64	64	74	72	64	61	71	72	62	63	62	61
Accuracy (%)																											
Older	95	93	96	96	88	95	93	91	96	94	94	96	96	92	97	94	92	98	93	94	97	93	88	96	91	93	97
SD	6	7	3	5	12	4	7	8	5	5	5	3	3	7	2	6	6	2	7	4	2	8	10	3	9	5	3
Younger	97	98	97	97	97	97	98	97	99	98	97	98	99	97	98	98	96	99	98	97	99	98	96	98	99	98	99
SD	3	5	3	3	4	3	2	4	1	3	3	2	2	2	2	2	3	2	3	3	2	2	3	2	2	2	1
False alarm (%)																											
Older	4	9	8	5	6	6	7	4	5	6	9	7	6	12	8	3	6	4	9	10	7	9	14	10	3	7	2
SD	8	10	9	8	9	10	10	6	9	11	9	11	7	8	9	6	8	8	9	13	10	12	14	11	6	12	5
Younger	4	4	4	4	5	8	2	0	1	2	8	5	1	8	6	1	2	2	4	8	5	3	9	6	1	1	1
SD	6	8	9	6	5	7	5	0	2	5	10	6	3	8	9	2	4	4	6	9	7	6	8	10	2	2	2

*Note*. A = auditory stimuli; V = visual stimuli; AV = audiovisual stimuli; *SD* = standard deviation.

### Response Time

The RTs to each stimulus under each cue condition was shown in Table 1. The Group (Older, Younger) × Cue Type (A_cue, V_cue, AV_cue) × Cue Validity (Valid, Invalid, No cue) × Stimulus Type (A, V, AV) ANOVA for RTs showed significant main effect of group—*F*(1, 38) = 14.77, *p* < .001, demonstrating a faster response by younger adults than by older adults, and main effect of cue validity—*F*(2, 76) = 246.26, *p* < .001, demonstrating the fastest response in valid-cue condition and lowest response in no-cue condition. In addition, a significant main effect of stimulus type was also identified—*F*(2, 76) = 96.82, *p* < .001, demonstrating a faster response to AV than A or V (AV > V > A). The interaction between cue modality and cue validity was significant—*F*(4, 152) = 9.10, *p* < .001, and the post hoc analysis showed that the response was the fastest in the valid cue condition (valid cue > invalid cue > no cue, all *p*s ≤ .001) for all cue modalities. For the valid- and no-cue conditions, no significant difference was found among cue modalities; however, for the invalid-cue trial, the response was faster in the visual-cue condition compared with the auditory-cue condition (*p* = .039). The interaction between cue modality and stimulus type was also significant—*F*(4, 152) = 4.82, *p* = .003, and the post hoc analysis showed that the response to AV was faster than A or V in all cue-modality conditions (AV > V > A, all *p*s < .001), and there was no significant difference among cue modalities for each cue type (all *p*s ≥ .085). Additionally, a significant interaction was noted between cue validity and stimulus type—*F*(4, 152) = 30.52, *p* < .001. Post hoc analysis showed that in all cue validity conditions, the response to AV was faster than A or V (AV > V > A, all *p*s ≤ .007). For the A stimulus, no significant difference was noted between valid- and invalid-cue conditions (*p* = .296), but a significantly faster response to valid or invalid cues compared with no-cue conditions (all *p*s < .001). For V and AV stimuli, the response was faster in valid-cue conditions (valid cue > invalid cue > no cue, all *p*s ≤ .001).

### Race Model Comparisons

**Figure 2.**
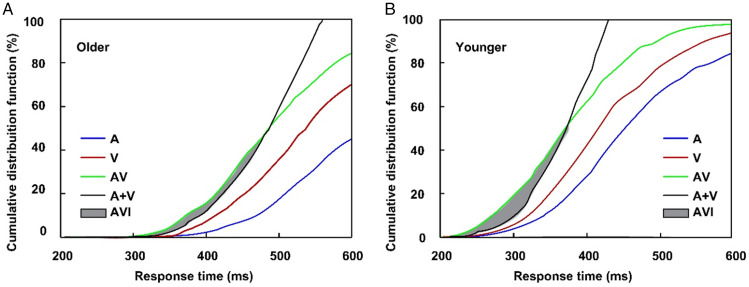
Cumulative distribution functions of response time for auditory stimuli, visual stimuli, audiovisual stimuli, and race model for older (A) and younger (B) adults in no-cue conditions. A = auditory stimuli; V = visual stimuli; AV = audiovisual stimuli; AVI = audiovisual integration.

In all cue-type conditions, no-cue trials were included. Given the lack of significant differences in the RTs, accuracy, false alarm, and AVI for no-cue trial among all cue-type conditions (all *p*s > .05), the data under visual cues, auditory cues, and audiovisual cues were averaged. The main aim of this study was to investigate the different effects of bimodal cues and unimodal cues on the AVI; therefore, only the AVI in the valid-cue conditions was applied for further analysis. The accuracy was greater than 90% and false alarm is lower than 10% under valid-cue and no-cue conditions for both older and younger adults (Table 1); therefore, the *kill-the-twin*-correction of false alarm for nontarget stimuli was employed according to the studies by Gondan and Minakata (2015, Ineq 10). The comparison of CDFs between AV and race model showed significant AVI in no-cue conditions for both older (270–320 ms, *t*_max_ = 7.57, *p* < .001, Figure 2A) and younger (250–360 ms, *t*_max_ = 8.19, *p* < .001, Figure 2B) adults. The significant AVI was only found in visual-cue condition (250–310 ms, *t*_max_ = 6.86, *p* = .014) for older adults, however, for younger adults, significant AVI was found in all of the visual- (230–350 ms, *t*_max_ = 6.24, *p* = .009), auditory- (230–340 ms, *t*_max_ = 5.25, *p* = .013), and audiovisual- (230–320 ms, *t*_max_ = 8.43, *p* < .001) cue conditions.

**Figure 3.**
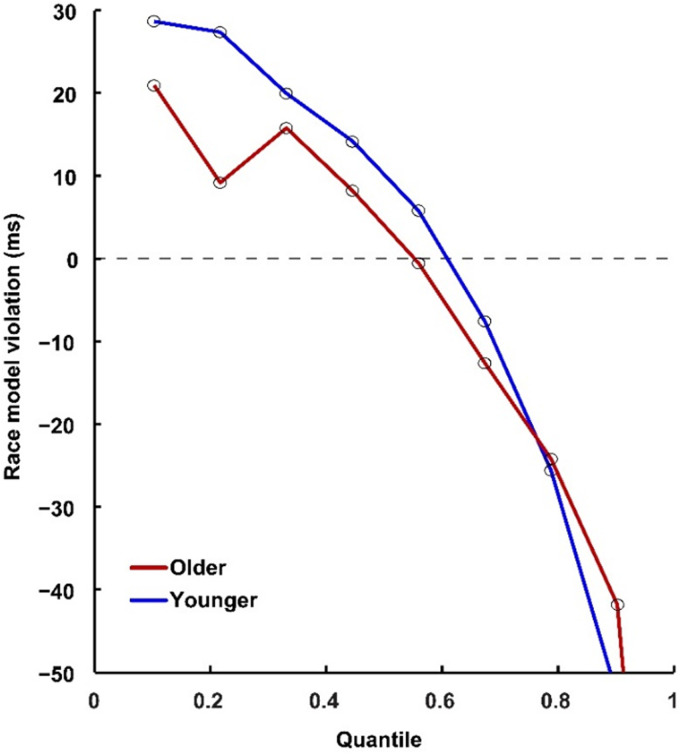
Race model violation for older adults (red line) and younger adults (blue line) in no-cue conditions.

**Figure 4.**
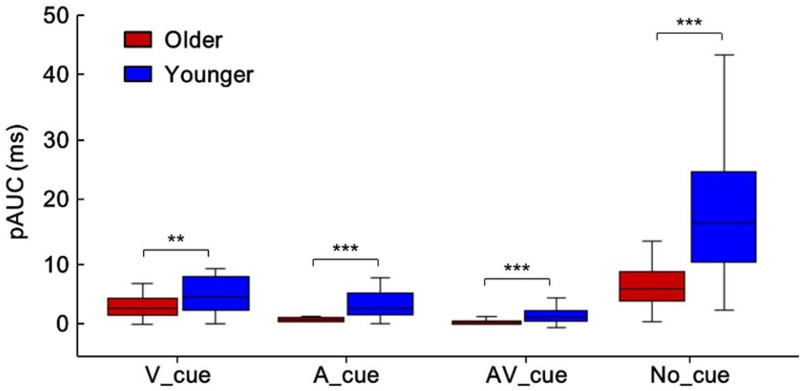
Higher pAUC for younger adults than for older adults in all cue-type conditions. **p < .01. ***p < .001. A = auditory stimuli; V = visual stimuli; AV = audiovisual stimuli; pAUC = positive area under violation curve.

To investigate the different effects of cue type on AVI, the average amount that violated race model inequality for each cue condition was computed, as shown in Figure 3 for no-cue conditions by older (red) and younger (blue) adults. Significant AVI was found in no-cue (6.6 ms) and visual-cue (2.9 ms) conditions, but not in auditory-cue and audiovisual-cue conditions for older adults. For younger adults, significant AVI was found in all cue conditions, with 4.6 ms, 2.9 ms, 1.8 ms, and 19.3 ms for auditory-, visual-, audiovisual-, and no-cue conditions, respectively. The Group (Older, Younger) × Cue Type (A_cue, V_cue, AV_cue, No_cue) ANOVA for pAUC showed main effect of group—*F*(1, 38) = 31.68, *p* < .001, showing that the pAUC was lower for older adults than for younger adults under all cue-type conditions, indicating a reduced AVI in older adults. Besides, significant main effect of cue type was also found—*F*(3, 114) = 52.60, *p* < .001, showing highest pAUC in no-cue condition (no cue > visual cue > auditory cue = audiovisual cue). The interaction between group and cue type was significant—F(3, 114) = 12.35, *p* < .001. The post hoc analysis showed higher pAUC for younger adults than for older adults under all cue type conditions (Bonferroni correction, all *p*s ≤ .004, Figure 4). Besides, the pAUC was highest under no-cue condition and lowest under audiovisual cue condition (no cue > visual cue > auditory cue > audiovisual cue, all *p* ≤ .041), and the pAUC was higher under no-cue condition than that under visual cue condition (*p* < .001).

## Discussion

The aim of this study was to investigate the effect of exogenous cue modality on AVI and its aging effect. The results showed that the AVI was lower in the exogenous-cue condition compared with the no-cue condition for both older and younger adults. For younger adults, the AVI was lower in the audiovisual-cue condition compared with the visual- and auditory-cue conditions; however, the AVI was only found in the visual-cue condition but not in the auditory- and audiovisual-cue conditions for older adults. In addition, the AVI was lower for older adults than for younger adults in no- and visual-cue conditions.

Consistent with previous studies (Van der Stoep et al., 2015, 2017; Xu et al., 2020), exogenous cues decreased the AVI. Previous studies have shown that exogenous cues facilitate the detection of stimuli presented at cued locations rather than no-cued locations by altering the resting state into a new state in preparation for detection and response to the following stimulus (Posner & Petersen, 1990, 2012). In this study, the participants were instructed to respond to the target as accurately and quickly as possible; therefore, the anterior cue alerts the emergence of a subsequent target, which leads to an enhanced perceptual sensitivity and a decreased perceptual threshold to the subsequent stimulus in the cued location than in the no-cued location (Lore et al., 2013). Therefore, in the cue trials, the participants’ perceptual intensity for identical stimulations might be stronger than in the no-cue trials. Consistent with the inverse effectiveness of AVI, stimuli with higher intensity inversely produce a lower AVI (Meredith et al., 1987; Stein & Meredith, 1993). Therefore, the lower AVI in the cued condition might be mainly attributed to the enhanced sensitivity resulting from the alerting effect.

The AVI was lower in the bimodal cue condition than in the unimodal cue condition for younger adults at 4.6 ms, 2.9 ms, and 1.8 ms for visual-, auditory-, audiovisual-cue conditions, respectively, which was consistent with our original hypothesis. Studies by Santangelo et al. (Santangelo & Spence, 2007; Santangelo et al., 2008; Spence & Santangelo, 2008) demonstrated that bimodal exogenous cues captured much higher and more stable cueing effects than unimodal cues; therefore, the cueing effect was higher in the bimodal cue condition than in the unimodal cue condition. In addition, both this study and previous investigations have confirmed that exogenous attention decreases the AVI (Van der Stoep et al., 2015, 2017; Xu et al., 2020). Therefore, it is reasonable for the lower AVI in the bimodal cue condition than in the unimodal cue condition. The nerve-center energy theory proposed by Kahneman and Tversky (1973) refers to the fact that the energy for each person is limited, and if one stimulus could expend more energy, the other stimuli could expend less energy (Kahneman and Tversky, 1973). The anterior cue shifted more center energy to the stimulus in the visual modality in the visual-cue condition but to the auditory modality in the auditory-cue condition. Visual dominance has been widely reported during the integration of auditory and visual information (Collignon et al., 2008; Gao et al., 2018; Paraskevopoulos et al., 2015; Sekuler et al., 1997), demonstrating that the AVI relies much more on the processing of visual clues for the integration of audiovisual information. Therefore, the AVI was higher in the visual-cue condition compared with the auditory-cue condition in the present study.

This study showed for the first time, to our knowledge, the aging effect of AVI in exogenous cue conditions, showing that the AVI was found in the visual-cue condition but not in the auditory and audiovisual-cue conditions. Studies on age-related attention found a significant deficit of distractor suppression in older individuals (Kok, 2000; Plude et al., 1994; Quigley et al., 2010), and older adults are much more easily disturbed by irrelevant distractors (Williams et al., 2016). In this study, the ratio of valid, invalid, and no cue trials was 1:1:1, so older adults had to select visual and auditory targets from masses of irrelevant distractors, such as auditory nontargets, visual nontargets, visual cues, and auditory cues. In addition, all the participants were required to respond to the target stimuli as accurately and quickly as possible, which also adds cognitive load (Kahneman & Tversky, 1973). To ensure a higher accuracy, more central energy was allocated to suppress the irrelevant distractor, which prevented the integration of information from different modalities simultaneously. Therefore, the most possible contributor to absent AVI in auditory- and audiovisual-cue conditions in older adults might be their attentional deficit. However, in the visual-cue condition, a slight AVI (2.9 ms) occurred. Visual dominance during the integration of auditory and visual information was also found (Diaconescu et al., 2013); therefore, slight AVI in older adults might also be attributed to the visual dominance effect. Besides, the results were similar with that of younger adults, showing lower AVI in the bimodal cue condition than in the unimodal cue condition. The similar pattern of results between older and younger adults might be mainly attributed that there is just more noise in the data of the older adults than in the young adults, which is the limitation of the present study, and need further imaging studies to clarify.

Additionally, in the no-cue condition, the AVI was lower in older adults compared with younger adults, which was consistent with the studies by Wu et al. (2012), Mahoney et al. (2011), Ren et al. (2016), and Ren, Li, et al. (2020). In the studies of Wu et al. and Ren et al., a similar AV discrimination task with black-white checkerboards and white noise/pure tone was applied. In their study, the visual, auditory, and audiovisual stimuli were presented in a random order, and the participant was instructed to respond to the target stimuli as accurately and quickly as possible. The race model analysis showed a relatively lower AVI for older adults than younger adults. In Mahoney et al.’s study, a detection task with asterisks and pure tones was used, and consistent results were obtained using the race model. However, controversial results were also found, revealing an enhanced AVI for older adults compared with younger adults using a race model (Laurienti et al., 2006; Peiffer et al., 2007). With aging, there is a serious decline in spatial information processing (Beurskens & Bock, 2012). In the studies of Wu et al., Mahoney et al., and Ren et al., visual and auditory stimuli were present peripherally but were presented centrally in the studies by Laurienti et al. and Peiffer et al. Therefore, the most likely reason for the contradictory results might be mainly attributed to the presented location for stimuli. The lower AVI was mainly attributed to the decline in peripheral information processing.

In conclusion, exogenous cues decreased the AVI, and the AVI was lower in the bimodal cue condition compared with the unimodal cue condition. In addition, for older adults, the AVI was reduced seriously due to their attentional deficit.
